# The effects of temperature variation treatments on embryonic development: a mouse study

**DOI:** 10.1038/s41598-022-06158-y

**Published:** 2022-02-15

**Authors:** Dóris Ferreira Moriyama, Dimitra Makri, Mary-Naya Maalouf, Petra Adamova, Gabrielle Ferrante Alves de Moraes, Marcela de Oliveira Pinheiro, Danilo Lessa Bernardineli, Irineu Francisco Delfino Silva Massaia, Walid E. Maalouf, Edson Guimarães Lo Turco

**Affiliations:** 1 Department of Surgery, Division of Urology, Human Reproduction Section, Medical School - EPM, Universidade Federal de São Paulo – UNIFESP, São Paulo, 04039-060 Brazil; 2 Division of Child Health, Obstetrics, and Gynaecology, East Block Queen’s Medical Centre, School of Medicine, University of Nottingham, Nottingham, NG72UH UK; 3 Institute of Physics, Universidade de São Paulo – IF USP, São Paulo, 05508-020 Brazil; 4 Faculdade de Ciências Médicas da Santa Casa de São Paulo, São Paulo, 01221-020 Brazil

**Keywords:** Biotechnology, Developmental biology, Health care

## Abstract

Since the development of ART, embryos have been cultured at 37 °C in an attempt to mimic the in vivo conditions and the average body temperature of an adult. However, a gradient of temperatures within the reproductive tract has been demonstrated in humans and several other mammalian species. Therefore, the aim of this study was to evaluate the effects of temperature variation treatments on mouse embryo quality through morphokinetic events, blastocyst morphology, the relative gene expression of *Igf2, Bax, Bcl2* and *Apaf1* and the metabolomics of individual culture media. Study groups consisted of 2 circadian treatments, T1 with embryos being cultured at 37 °C during the day and 35.5 °C during the night, T2 with 38.5 °C during the day and 37 °C during the night and a control group with constant 37 °C. Our main findings are that the lower-temperature group (T1) showed a consistent negative effect on mouse embryo development with “slow” cleaving embryos, poor-quality blastocysts, a higher expression of the apoptotic gene *Apaf1*, and a significantly different set of amino acids representing a more stressed metabolism. On the other hand, our higher-temperature group (T2) showed similar results to the control group, with no adverse effects on blastocyst viability.

## Introduction

The fundamental objectives of ART are offering patients the best possible live birth of healthy offspring while avoiding multiple pregnancies and complications such as OHSS. Although the issue of multiple pregnancies has significantly decreased after the implementation of the single-embryo transfer (SET) policy in a number of countries, IVF treatments are still challenged by many difficulties, such as moderately low implantation rates. The key factors for a successful implantation are endometrial receptivity, a competent blastocyst and the synchronicity between them, which will guarantee the connexion between embryo and maternal tissue^1–3^. Among these key factors that will lead to a successful implantation this paper will focus on the quality of the blastocyst. It is known that several physical and chemical factors within the ART laboratory may affect embryonic development and viability, such as culture media composition, plasticware, oil overlay, volatile organic compounds, gas tension and pH^4–6^. Among the physical factors, temperature has been neglected. The reason behind the use of 37 °C for human cell culture in vitro is an attempt to mimic the in vivo conditions, as the normal body temperature of an adult is scientifically accepted worldwide as 37 °C. However, studies conducted mainly with animal models have shown a gradient of temperatures within the female reproductive tract^7–13^. In rabbits, for example, the temperature inside the ovarian follicle is 1.4 °C cooler than its deep body temperature^11^ and a rise in temperature is observed when ovulation approaches^13,14^. Other studies revealed that mature Graafian follicles in rabbits and pigs are not only cooler than the body temperature but also cooler than the rest of the stroma in ovaries, demonstrating temperature variation within this organ^7,12^. Moreover, the basal body temperature of women has long been associated with the menstrual cycle, timing of ovulation and natural family planning due to the thermogenic properties of the progesterone^15,16^. During the luteal phase of the menstrual cycle, a rise of 0.31–0.46 °C occurs in the basal body temperature of women^17,18^. The study of Ng, K et al*.* (2018)^13^ proposes several factors that might influence temperature fluctuation in the female reproductive tract, such as metabolic activity and the rate of heat loss within the organs, depending on its proximity with other internal body structures, as well as its muscle and blood vessel composition. The same authors also brilliantly stated that many female causes of infertility, such as endometriosis, obesity and polycystic ovarian syndrome, probably alter the temperature gradient in the reproductive axis^13^.

Since very early in the history of ART, embryo morphology has been the principal method to assess and select the best embryo prior to transfer^19–21^. With the advent of time-lapse systems, the assessment of embryo morphology was then analysed dynamically over time, providing more insights into embryo growth^22–24^. However, morphological assessments will not necessarily reflect DNA stability in the embryo or its transcription capabilities. Therefore, this study aimed to investigate the effects of temperature variation treatments on mouse embryo quality through morphokinetic events and blastocyst morphology. Additionally, blastocyst quality was also evaluated individually at the molecular level through the relative gene expression of the following stress and apoptotic genes: Igf2, Bax, Bcl2 and Apaf1 and through the targeted metabolomics of culture media, focusing on the main amino acids involved in preimplantation embryonic development. The Igf2 gene is commonly expressed during murine embryonic development, playing an important role in placental growth^25,26^. Overexpression of this gene is associated with human embryonic stress and potential foetal overgrowth^27^. Furthermore, in studies aiming to understand possible epigenetic consequences in IVF babies, the Igf2 gene is known to be a biomarker for mouse and human models^28,29^. The apoptosis pathway consists of several genes in a cascade, leading to DNA fragmentation and cell death. At the beginning of this pathway, Bax and Bcl2 play an important role. While Bax is a proapoptotic gene that triggers the cell death response; Bcl2 is antiapoptotic, preventing mitochondrial changes and activation of the caspase pathway, thus promoting cell survival^30–32^. On the other hand, Apaf1 is an effector member of the apoptosis cascade and is responsible for stimulating caspases, which will then promote chromatin condensation, DNA fragmentation and cell surface alteration^30,33^. Based on the existing knowledge, the hypothesis of the present study is whether temperature variation treatments impair the developmental competence and quality of mouse embryos.

## Materials and methods

### Study groups

To build the study groups, first, it was considered the premise of the circadian cycle of humans and other mammalian species, which are active during the day and inactive during the night or vice versa. Second, bearing in mind that temperature variation treatment may potentially benefit human embryonic development in vitro, the temperature variation treatments tried to mimic the slight decrease in body temperature during rest. Therefore, warmer temperatures were always used during the day (12 h, from 9 am to 9 pm), while cooler temperatures were applied during the night (12 h, from 9 pm to 9 am). Additionally, a maximum interval of 2 °C above or below 37 °C was used for treatments, as previous studies have shown the existence of a similar gradient of temperature within the female reproductive tract (ovaries, fallopian tubes and uterus) in several mammalian specie^7,8,11,12^. Studies have also considered heat stress at temperatures above 39 °C^34–36^ or detrimental effects below or at 35 °C^37,38^. Thus, the study groups in the present study were as follows: Treatment 1 (T1), with mouse embryos being cultured the from zygote stage to the blastocyst stage at 37 °C during the day and 35.5 °C during the night; Treatment 2 (T2), with 38.5 °C during the day and 37 °C during the night; and the Control group (C), with a constant temperature of 37 °C.

### Study design

A total of 161 frozen-thawed mouse blastocysts of a hybrid strain were used to conduct this study. The embryos were cultured in EmbryoScope time-lapse incubators (Vitrolife, Göteborg, Sweden), with 53 from the T1 group, 53 from the T2 group and 55 from the C group. Embryo quality was assessed through morphokinetic events, from embryo’s first cleavage to hatching of the blastocyst, along with morphological assessment. When embryo culture was terminated (96 h after thaw), the blastocysts and droplets of culture media were collected individually. Out of the 161 embryos that reached the blastocyst stage, 83 blastocysts were used for the relative gene expression analysis and the corresponding media of these 83 blastocysts were used for metabolomics analysis. The experiments were performed in the School of Medicine, Division of Child Health, Obstetrics, and Gynaecology, East Block Queen’s Medical Centre, at the University of Nottingham (United Kingdom) and were exempt from ethical approval, as frozen embryos were purchased and cultured up to the blastocyst stage according to the Animals Scientific Procedures Act 1986 (ASPA) for the protection of animals.

### Mouse embryo thawing and time-lapse culture

**Figure 1:**
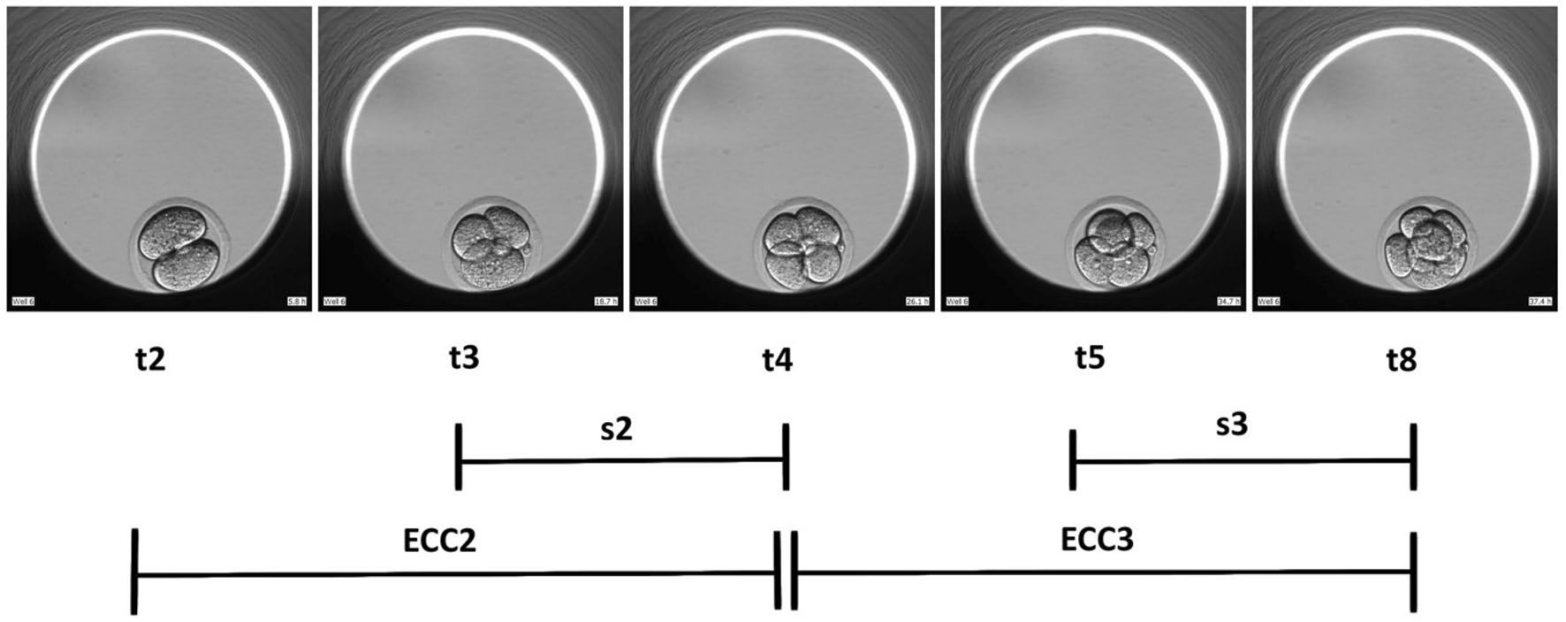
Time-lapse snapshots of early mouse embryo development. The t2, t3, t4, t5 and t8 all relative to tPNf are depicted, as well as, the calculations for synchronicity (s2, s3) and duration of second and third cell cycles (ECC2, ECC3). Photos from the experiments used to build this figure were collected using the EmbryoViewer software.

**Figure 2:**
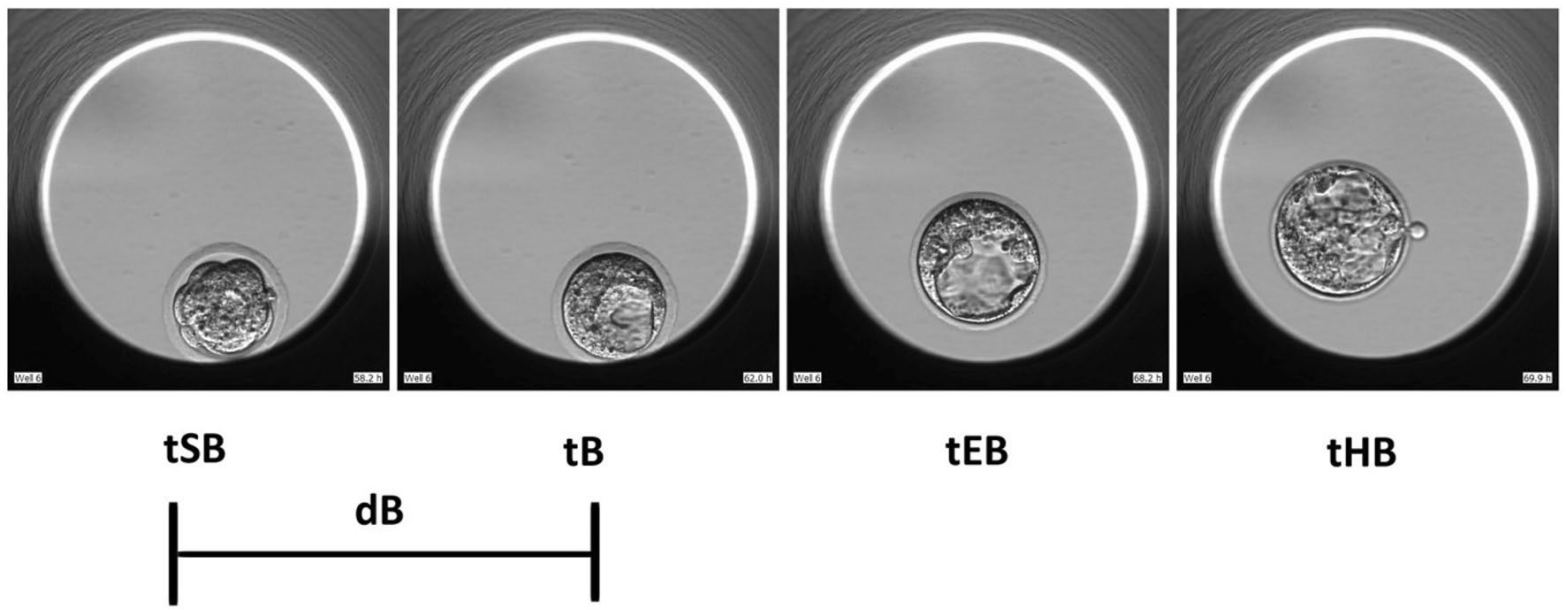
Time-lapse snapshots of the mouse embryo development post-compaction. The tSB, tB, tEB and tHB all relative to tPNf are depicted, as well as, time of blastulation (dB). Photos from the experiments used to build this figure were collected using the EmbryoViewer software.

Mouse embryos from a hybrid strain (B6C3F1 x B6D2F1) obtained from Embryotech Laboratories (MA, USA) were used for all experiments and the 2 strains were matched in each of the individual study arms. Mouse embryo thawing and culturing was performed in 3 consecutive repetitions of the experiment. On Day 1, 1-cell stage embryos were thawed in HEPES buffered medium (Sigma-Aldrich, UK) following the manufacturer’s protocol (https://embryotech.com). The embryos were individually placed in microwells of EmbryoSlides (Vitrolife, Göteborg, Sweden) previously prepared with continuous single culture complete media (CSCM-C) with gentamicin sulfate and human serum albumin (Irvine, Santa Ana, USA), overlaid with 1.4 ml of EmbryoMax filtered light mineral oil (Merck, UK) and equilibrated at 37 °C and 6% CO_2_. The embryos were cultured in the EmbryoScope time-lapse incubator (Vitrolife, Göteborg, Sweden) at 6% CO_2,_ atmospheric O_2_ and dry atmosphere under 3 temperature variation treatments: T1 with 37 °C and 35.5 °C, T2 with 38.5 °C and 37 °C and C with constant 37 °C. Temperature validation was performed prior to data collection using validated thermometers and gas analysers. To carry out temperature variation treatments, 3 EmbryoScopes were used (one for each study group) and the temperature was changed in the machines set up at 9 am and 9 pm. Each embryo was captured with an in-built camera every 15 min at 9 focal planes. Experiments terminated at 9 am of day 5 (96 h post-thaw), and only embryos that reached the blastocyst stage (N = 161) were considered for further analysis. EmbryoViewer software (Vitrolife, Göteborg, Sweden) was used to process data and according to previous guidelines^39^, the following morphokinetic events were annotated: first cleavage (t2), division into 3-cell (t3), 4-cell (t4), 5-cell (t5) and 8-cell (t8) stages, start of blastulation (tSB), full blastocyst (tB), expanded blastocyst (tEB) and hatching of the blastocyst (tHB) (Figs. 1 and 2, Supplementary material – Table I). All time points were normalized to pronuclear fading (tPNf) to maintain consistency among embryo annotations and avoid bias due to the time of fertilization^40,41^. Using this information, the durations of the second (ECC2 or t4-t2) and the third cell cycles (ECC3 or t8-t4), as well as, the synchronicity of the second (s2 or t4-t3) and third cell cycles (s3 or t8-t5), and the duration of blastulation (dB) were calculated (Figs. 1 and 2 and Supplementary Material – Tables I and II). Additionally, the ratios of the cleavage synchronicities from 2 to 4 cells (CS2-4), 2 to 8 cells (CS2-8), and 4 to 8 cells (CS4-8) were calculated as previously described by^42^. Finally, the blastocysts were morphologically graded as poor (1), fair (2) or good (3) at the specific time frame of 90.0 h (post-thaw) using the methodology proposed by Gardner et al. (2000)^43^ and the Society for Assisted Reproductive Technology (SART)^44^.

### cDNA production and qPCR

After 96 h of culture, each individual blastocyst was collected in a minimum volume of culture media from EmbryoSlide, placed in a 0.2 ml microtube, immediately snap-frozen in liquid nitrogen, and kept at -80 °C until qPCR analysis. For the relative gene expression analysis, 24 blastocysts from the T1 group, 30 from the T2 group and 29 from the C group were used (N = 83). RNA samples from individual blastocysts were isolated using a RNeasy Micro Kit (Qiagen, Manchester, UK) based on instructions provided by the manufacturer. cDNA templates were built using the High-Capacity RNA-to-cDNA kit (Applied Biosystems, UK), according to guidelines provided by the supplier. Quantitative PCRR (qPCR) was carried out using TaqMan Gene Expression Assays (Applied Biosystems, UK) to amplify Igf2, Bax, Bcl2 and Apaf1 in a duplex reaction with Rpl5. The samples were run in duplicate for each gene of interest in the 7500 Fast Real-Time PCR System (Applied Biosystems, UK) under the following cycling conditions: 50 °C holding stage for 2 min, 95 °C holding stage for 20 s, followed by 40 cycles of 95 °C for 3 s and 60 °C for 30 s. All data were analysed with 7500 software V2.3 (Applied Biosystems, UK) using Ct values as read-outs. The Rpl5 was the housekeeper gene used for normalizing the Ct values and calculating the ΔCt^45^. A Ct-value of 35 was used as the cut-off to capture all potentially valid signals. The mean fold change between study groups was calculated following the 2^-ΔΔCt^ method^45^.

### Targeted metabolomics

Approximately 25 µl of culture media was collected from each well of the Embryoslides, and the droplets collected were then snap frozen in liquid nitrogen and stored at -80 °C for targeted metabolomics. The following amino acids were identified and quantified: phosphoserine (PHSER), taurine (TAUR), phosphoethanolamine (PEA), urea (UREA), L-aspartic acid (ASP), L-threonine (THR), serine (SER), L-asparagine (ASN), L-glutamic acid (GLU), glutamine (GLN), L-sarcosine (SARC), glycine (GLY), L-alanine (ALA), citrulline (CITR), valine (VAL), cystine (CYS), L-methionine (MET), cystathionine (CYSTH), L-isoleucine (ILE), L-leucine (LEU), norleucine (NLEU), tyrosine (TYR), β-alanine (B-ALA), phenylalanine (PHE), homocystine (HOMOCYS), ethanolamine (ETHAMN), ammonium chloride (AMM), D-allohydroxylysine (HYLYS 1), L-allohydroxylysine (HYLYS 2), L-ornithine (ORN), L-lysine (LYS), 1-methyl-L-histidine (HIS), tryptophan (TRP), 3-methyl-L-histidine (3-mhis), anserine (ANS), L-carnosine (CAR), L-arginine (ARGINE), serotonin (SEROT) and L-proline (PRO). As internal standards for quantification 4 amino acids that were inert, nontoxic and unmodifiable by the embryos were used: L-α-aminoadipic acid (aaaa), α-aminobutyric acid (aaba), β-aminoisobutyric acid (baiba) and γ-aminobutyric acid (gaba). Out of the 83 samples, including the blastocysts with the corresponding culture media droplets, only 81 droplets were used for metabolite extraction due to a volume limitation, being 24 from the T1 group, 29 from the T2 group and 28 from the C. For this analysis, 9 samples were collected from the washing microwells of the EmbryoSlides as negative controls, 3 from each study group. Collection of “blank droplets”, which meant that the droplets of culture media without embryos from the EmbryoSlides were essential for this analysis. With these negative control samples, it was possible to distinguish the embryonic metabolome from culture media degradation, resulting in the turnover profile of each amino acid. Identification followed by quantification of targeted metabolites was carried out using Biochrom 30 equipment (Biochrom Limited, UK), which consists of HPLC coupled to a spectrophotometer. The extraction of the amino acids was performed according to the manufacturer’s instructions.

### Statistical analysis

SPSS Statistics 24.0 software (IBM, USA) was used for most variables, except for the metabolomics data. Categorical variables, such as morphological assessment of the blastocysts, were analysed by chi-square test. Numerical variables were tested for normality with the Shapiro–Wilk test; if not normally distributed, variables were then standardized by the *zescore*. Continuous variables were then compared between groups using a general linear model (GLM,) with Bonferroni post hoc tests. Statistical difference was considered when p < 0.05. Graphs were generated using GraphPad Prism 8.1.2 software. Metabolomics data were analysed using the R 0.4, MixOmics 6.12.1 and Bioconductor 3.11 software. To evaluate the effects of temperature variation on the expression of metabolites during murine embryonic development, a partial least square discriminant analysis (PLS-Da) was performed. From the PLS-Da model, variables of importance in projection (VIPs) were determined in each study group. Finally, to check the effectiveness of the classification test, a receiver operating characteristic (ROC) curve was used.

## Results

### Morphokinetics and the morphological assessment of the blastocysts

**Figure 3:**
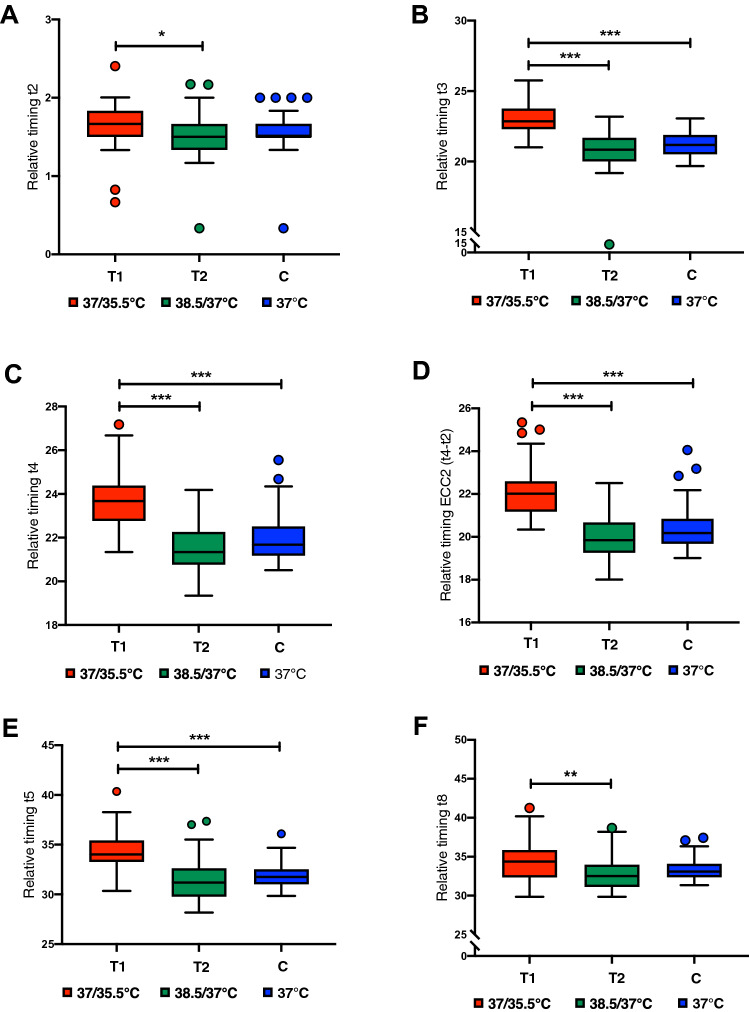
Box plots of early embryo development indicating the relationship between the relative timings and temperature variation treatments, (**A**) t2 (*P* = 0.039), (**B**) t3 (*P* = 0.0001), (**C**) t4 (*P* = 0.0001), (**D**) ECC2 (*P* = 0.0001), (**E**) t5 (*P* = 0.0001), (**F**) t8 (P = 0.007). Whiskers extend down to the minimum value and up to the maximum value, statistical difference between groups is marked with asterisks (*p < 0.05, ** p < 0.01 and ***p < 0.001). This figure was built using GraphPad Prism version 8.1.2 for Mac OS X, GraphPad Software, San Diego, California, USA, www.graphpad.com.

**Figure 4:**
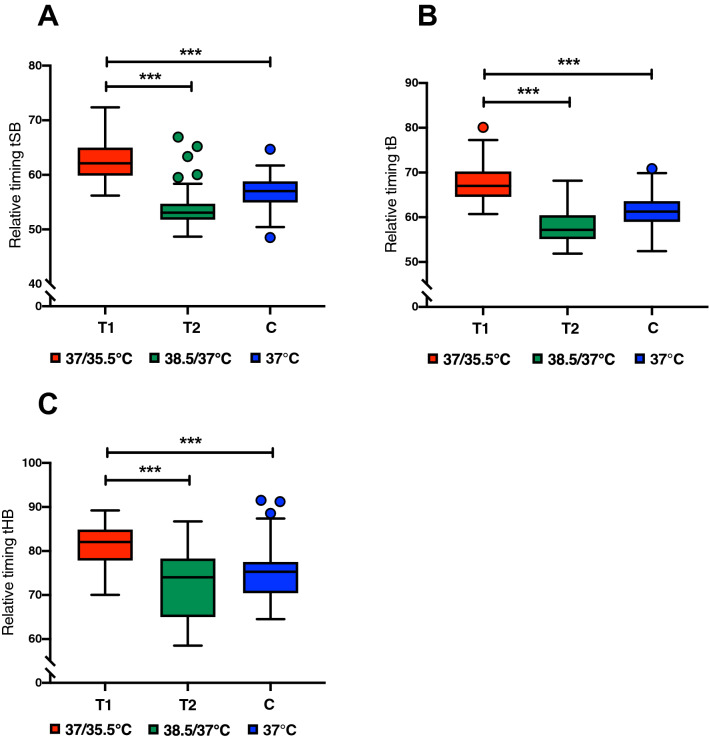
Box plots of embryo development post-compaction indicating the relationship between the relative timings and temperature variation treatments, (**A**) tSB (*P* = 0.0001), (**B**) tB (*P* = 0.0001), (**C**) tHB (*P* = 0.0001). Whiskers extend down to the minimum value and up to the maximum value, statistical difference between groups is marked with asterisks (*p < 0.05, **p < 0.01 and ***p < 0.001). This figure was built using GraphPad Prism version 8.1.2 for Mac OSX, GraphPad Software, San Diego, California, USA, www.graphpad.com.

**Figure 5:**
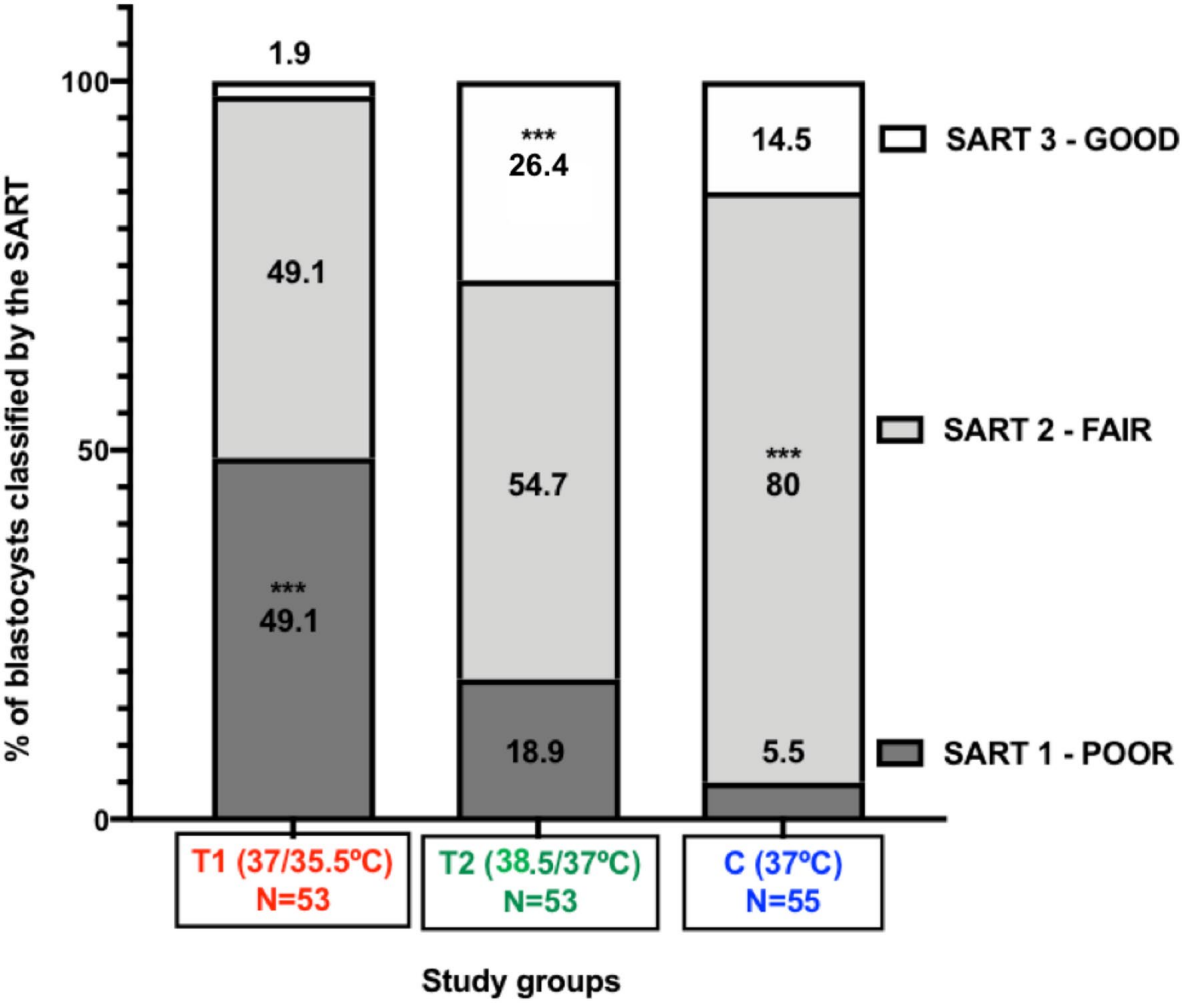
Morphological Assessment stack plots, each shade of grey represents the percentage of blastocysts scored as 1 (poor), 2 (fair) or 3 (good) in all study groups. Statistical difference between groups is marked with asterisks (*p < 0.05, ** p < 0.01 and ***p < 0.001). This figure was built using GraphPad Prism version 8.1.2 for Mac OS X, GraphPad Software, San Diego, California, USA, www.graphpad.com.

Data from 161 blastocysts were analysed to examine the effects of temperature variation treatments on morphokinetic events of embryo development before implantation. Regarding the early embryonic development, differences were found in the 2-cell (t2), 3-cell (t3) and 4-cell (t4) stages, the duration of the second cell cycle (ECC2 or t4-t2), and the 5-cell (t5) and 8-cell (t8) stages (Fig. 3, Supplementary Material – Table III and Video A). For all these variables, the T1 group showed a higher mean of relative timings, which indicates a slower development of the embryos cultured at 37/35.5 °C when compared to T2 (38.5/37 °C) and C (37 °C). Regarding embryonic development postcompaction, differences in the tSB, tB and tHB were found (Fig. 4, Supplementary Material – Table III and Video B). Following the same pattern observed at early embryonic development, embryos from the T1 group reached the early, full and hatching blastocyst stages later than the embryos from both groups T2 and C. Regarding the morphological assessment of the blastocysts (Fig. 5), generally the lower-temperature group (T1) resulted in poorer graded blastocysts with the highest percentage of blastocysts scored as poor (49.1%) when compared to the higher-temperature group (18.9%) and control (5,5%), with p = 0.0001. On the other hand, the higher-temperature group (T2) showed the highest percentage of blastocysts scored as good (26.4%), when compared to both the T1 (1.9%) and C (14.5%) groups, with p = 0.0001. The control group, as expected, was marked by a larger percentage of blastocysts scored as fair (80%) and the lowest percentage of blastocysts scored as poor (5.5%).

### Relative gene expression

**Figure 6:**
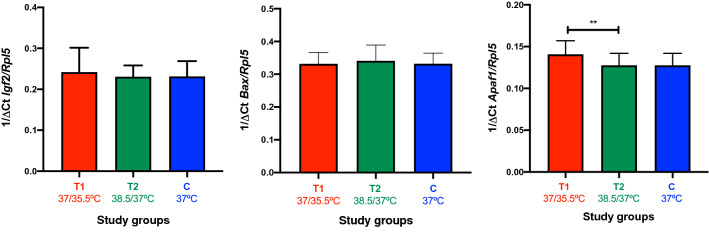
Bar charts represent mean of 1/ΔCt for the targeted genes detected in single blastocysts in each study group. Statistical difference between groups is marked with asterisks (*p < 0.05, ** p < 0.01 and ***p < 0.001). This figure was built using GraphPad Prism version 8.1.2 for Mac OS X, GraphPad Software, San Diego, California, USA, www.graphpad.com.

The relative expression of the Igf2, Bax, Bcl2 and Apaf1 genes of the blastocysts (N = 83) was used to analyse apoptosis and stress levels of the mouse embryos cultured in vitro under temperature variation treatments. The Bcl2 gene was undetectable for single blastocyst samples. For Igf2 and Bax, no statistically significant differences were found between the groups (Fig. 6). Nonetheless, differential expression analysis showed that the Apaf1 gene was significantly (*P* = 0.003) more abundant in the blastocysts cultured at 37/35.5 °C (T1 group with ΔCt 7.19 ± 0.16) than in blastocysts cultured at 38.5/37 °C (T2 group with ΔCt 7.93 ± 0.16) (Fig. 6 and Supplementary Material – Table IV), with a fold change of 1.67. Graphs were built based on 1/ΔCt for a direct relationship between relative gene expression and the study groups.

### Targeted metabolomics

**Figure 7:**
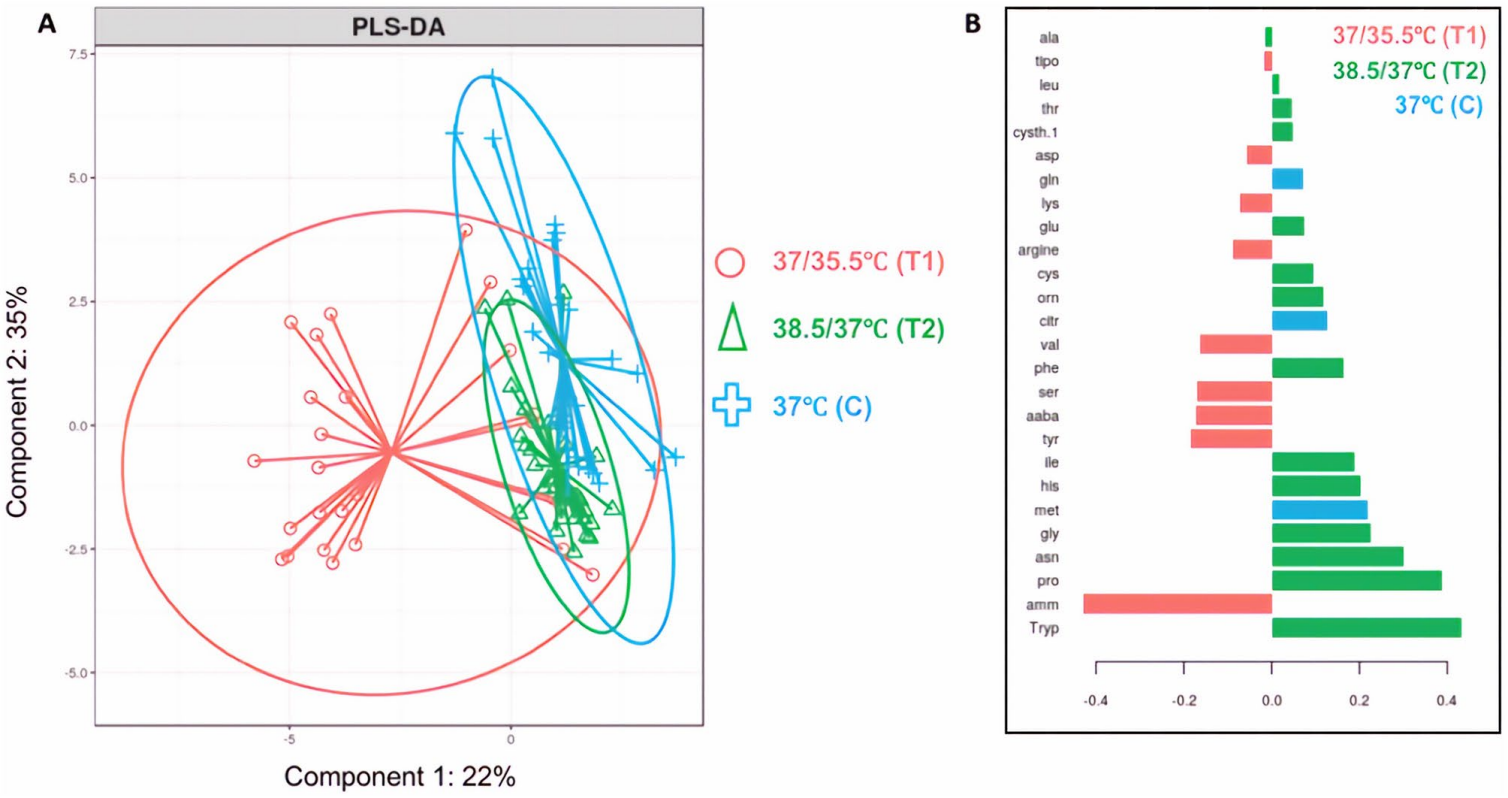
(**A**) Metabolites separated by study group in the partial least square discriminant analysis (PLS-Da). (**B**) The metabolites that contributed the most for the PLS-Da model (VIPs) are represented for each study group in the chart, while negative values indicate a negative correlation, the positive values indicate a positive correlation between the abundancy of the metabolites and the study groups. Graphs from this figure were built using mixOmics R package from Kim-Anh Le Cao, Florian Rohart, Ignacio Gonzalez, Sebastien Dejean with key contributors Benoit Gautier, Francois Bartolo, contributions from Pierre Monget, Jeff Coquery, FangZou Yao and Benoit Liquet. (2016). mixOmics: Omics. Data Integration Project. R package version 6.1.1. https://CRAN.R-project.org/package=mixOmics.

**Figure 8:**
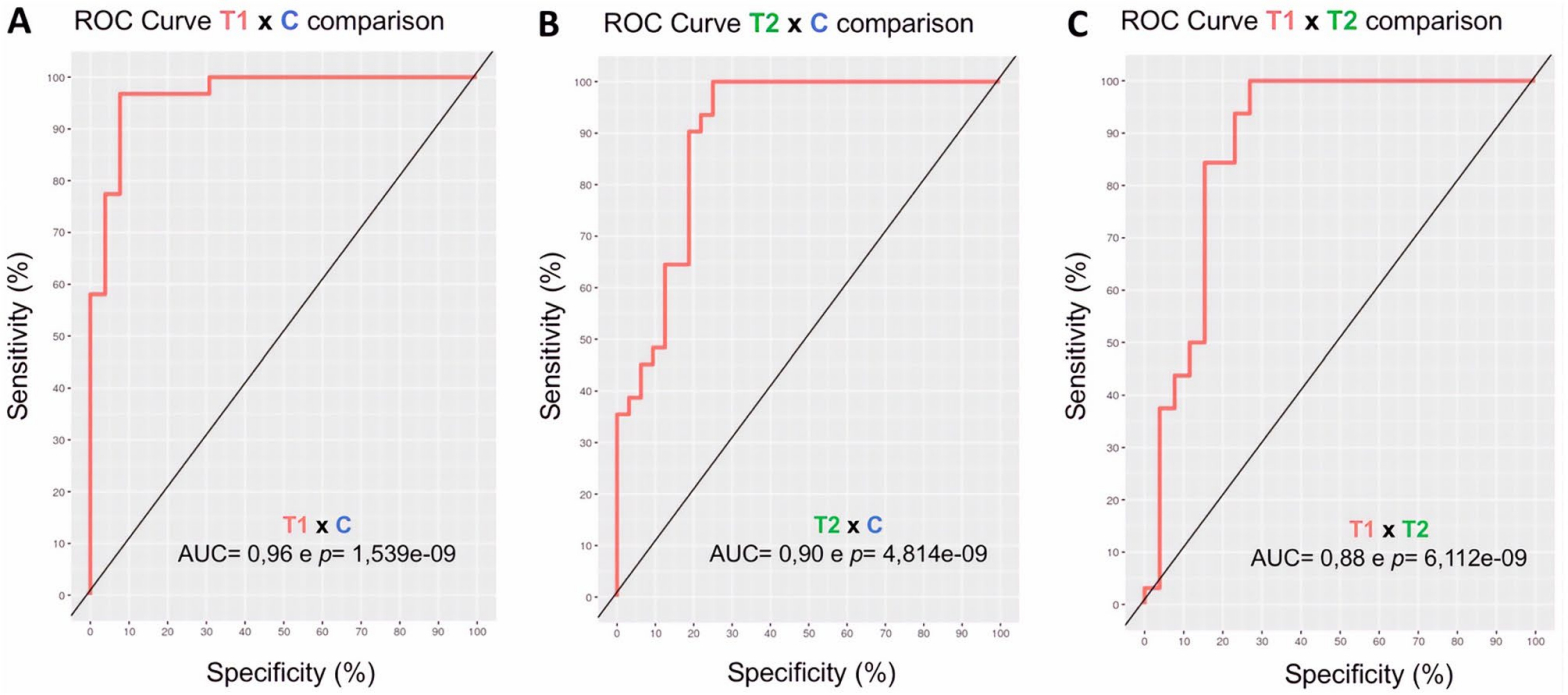
Receiver operating characteristic (ROC) curves showing the achieved sensitivity and specificity of the PLS-Da model in the comparisons: (**A**) lower-temperature group (T1) x control (C). (**B**) higher-temperature group (T2) x control (C). (**C**) lower-temperature (T1) x higher-temperature (T2). Graphs from this figure were built using mixOmics R package from Kim-Anh Le Cao, Florian Rohart, Ignacio Gonzalez, Sebastien Dejean with key contributors Benoit Gautier, Francois Bartolo, contributions from Pierre Monget, Jeff Coquery, FangZou Yao and Benoit Liquet. (2016). mixOmics: Omics. Data Integration Project. R package version 6.1.1. https://CRAN.Rproject.org/package=mixOmics.

PLS-Da (N = 81) was able to separate the metabolites according to each study group (Fig. 7). With this first step result, a clear separation was observed in the set of metabolites found for the lower-temperature group, when compared to both the higher-temperature and control groups. Throughout the VIP analysis, we showed that the set of amino acids that characterized the lower-temperature group was indeed different from the sets of the other 2 groups. In contrast, the set of amino acids that characterized the higher-temperature and control groups showed similarities, with some metabolites appearing in both groups. The ROC curves validated the statistical model, showing an area under the curve (AUC) of 96% (p = 1.539e-09) in the comparison between T1 x C, 90% (p = 4,814e-08) in the comparison of T2 x C and 88% (p = 6,112e-08) for T1 x T2 (Fig. 8). Interestingly, while the lower-temperature group set of metabolites indicated stressed embryonic metabolism, the other 2 groups were characterized by amino acids that play important roles during the metabolism of a normal/healthy preimplantation embryo.

## Discussion

Although other recent studies have investigated the effects of temperature variations on human mouse embryos^46–49^, to the best of our knowledge, this is the first study to deeply investigate the effects of temperature variation for 12 h on in vitro embryo development and quality through morphokinetics and morphodinamics, relative gene expression and metabolome profiles. The main findings of this study are that the temperature variation treatment of 37 °C during the day 35.5 °C during the night (T1), had a negative impact on mouse embryo development, affecting the embryonic morphokinetics pre and post compaction. At the molecular level, the negative impact of the T1 group was shown on higher expression of the Apaf1 gene in blastocyst cells and on stressed embryo metabolism. In contrast, the embryos that were cultured at 38.5 °C during the day and 37 °C during the night (T2) showed no adverse effects on embryonic development or embryo quality, with results always similar to those found in the control group.

To understand in-depth the effects of temperature variation treatments on mouse embryo quality, the morphokinetic events of embryonic development were assessed. The application of time-lapse monitoring enlightens the embryonic morphological assessment once it can detect potential abnormalities and/or dynamic changes in the features of the developing embryo during in vitro culture. As described by Wolff et al. (2013)^50^, the evaluation of mouse embryo morphokinetics has been considered a valid method for the quality control of in vitro culture and a useful marker of stress. Moreover, even though the use of time-lapse systems in the ART field has been a topic of great discussion in the scientific community^51–54^, human embryo selection through morphokinetics criteria has been associated with an improvement in ongoing clinical pregnancy and a decrease in early pregnancy loss^23^. Regarding early embryo development (precompaction), the present study found significant differences in the timings of the 2-cell (t2), 3-cell (t3), 4-cell (t4) and 5-cell (t5) stages, the duration of the second cell cycle (t4-t2 or ECC2) and the 8-cell (t8) stage. For the postcompaction stages, we found significant differences in the timings of small blastocoel (tSB), full blastocyst (tB) and hatching blastocyst (tHB). For all the morphokinetic parameters with statistically significant results, the cells of the embryos from the T1 group (37/35.5 °C) took longer to cleave and achieved further developmental stages when compared to the other two study groups, T2 (38.5/37 °C) and C (37 °C). Thus, we characterized the embryos of the T1 group as “slow” cleaving embryos, while the embryos from the T2 and C groups were considered “normal”. Throughout early embryo development, a cell undergoes a range of events in which duplication occurs, leading to division into two individual cells, known as the cell cycle. The duration between each cycle is the time needed for cytoplasmic cleavage, thus extended cell cycles may be caused by cellular rearrangement and probably DNA repair, before cleavage of the blastomeres^39,55^. A correlation between early cleavage parameters and the development and/or prediction of the blastocyst was reported in several other studies in both humans and mice^22,41,56–59^. Our study corroborates these findings, as early cleavage timings such as t3, ECC2, t4 and t5 were ranked as the most important variables for the statistical model (GLM), as evidenced by the effect size of those variables (0.44, 0.43, 0.42 and 0.36, respectively) (Supplementary Material—Table III). Additionally, external factors that directly affect culture conditions, such as oxygen levels^60,61^ and culture media composition^62^ have been reported to alter morphokinetic parameters, supporting our hypothesis that, like gas tension and solid embryo culture temperatures^63^, temperature variation treatments might also affect embryo morphokinetics.

Even with the recent development of several noninvasive methods to assess embryo quality, such as proteomics and/or metabolomics of spent culture media, analysis of specific microRNAs or embryo morphokinetics, embryo morphology alone is still largely used for embryo selection before transfer in many IVF centers worldwide. This practice is based on a well-established correlation between blastocyst quality and implantation followed by pregnancy^64–69^. Moreover, after the development of extended embryo culture the morphological assessment was then focused on the features of the blastocyst: the blastocoel, the inner cell mass and the trophectoderm. For the last 20 years, the scoring system proposed by Gardner et al. (2000)^43^ and the SART^70^ to evaluate blastocyst morphology on human embryos has not only been proven effective^66,68,71,72^, but also uncontested. Although the embryonic scoring system used in the present study was developed for humans, morphologically, these mammalian embryos are very similar, with the main morphological difference being the sizes of the embryos in these two species. Additionally, it is important to highlight that the mouse embryo bioassay has been one of the foundations of ART research and development and is an important tool for the quality control of in vitro embryo culture^57,73–76^. Therefore, in the present study, we found that mouse blastocysts cultured at 37 °C during the day and 35.5 °C during the night (T1), had a significantly lower score than blastocysts that were cultured at 38.5 °C during the day and 37 °C during the night (T2) and at a constant 37 °C (C).

Physical and chemical factors impact embryo development and blastocyst quality, and a minor alteration in one of these factors may result in a negative effect on embryo viability, followed by implantation failure^5^. Consequently, it was expected that the lower-temperature study group would result in a higher number of poorly graded embryos. In the study of Minasi *et* al. (2016)^71^, a correlation was found between the likelihood of euploidy, with higher blastocyst morphology scores and faster development after compaction (morphokinetic parameters), when embryos were cultured at the standard temperature of 37 °C. This study corroborates our findings, as the “normal” cleaving embryos (T2 and C) also had higher morphological scores than the “slow” cleaving embryos that belonged to the low-temperature group (T1), which had lower morphological scores at the blastocyst stage. Nonetheless, the morphological assessment of embryos can be biased by operator subjectivity, and ploidy in the cells has been proven deficient, which consequently affects the odds of successful implantation^77,78^. Thus, it is important to perform other analyses to assess embryo viability at the molecular level.

The development of human and other mammalian species embryos is associated with large amounts of cell proliferation and differentiation accompanied by apoptosis, which removes abnormal cells from the embryos as a self-correction mechanism. As the embryo develops, the apoptotic rate increases and more cells are eliminated. Apoptosis is more evident during late embryonic development, after embryo compaction and the start of blastulation^79^. More specifically, when the inner cell mass is then separated from the trophoblast, a process that end up with the death of cells that used to hold back together these two differentiated embryonic cell types^79–82^. Once isolated, the ICM and TE will also undergo apoptosis^83^, and even though cell death plays an important role in normal embryo development, under suboptimal conditions apoptosis is increased, resulting in DNA fragmentation and arrested embryo development^84^.

It is believed that throughout embryo development, the elimination of abnormal cells is associated with the expression pattern of specific apoptotic genes in the embryo^83^, which can be altered by either its activation or inhibition in response to both internal and external factors^6^. Among the internal factors are the cell machinery of the embryo itself and apoptosis. Many external factors have been shown to significantly impact the efficiency of the culture media, such as temperature, gas tension, Ph and osmolarity^85^. Together, these internal and external factors may influence the phenotype of embryos fertilized and cultured in vitro in the typical routine of an IVF laboratory^85^. The present study showed that the apoptotic peptidase activating factor 1 (Apaf1) was significantly more abundant in the blastocyst samples of group T1 (37/35.5 °C) than in those of group T2 (38.5/37 °C). Apaf1 is a protein-coding gene and a key factor in the apoptosis cascade^86^. This gene is particularly involved in the formation of the apoptosome, a ring-like molecule responsible for the activation of caspases, which leads to cell death^87^. Moreover, the expression of apoptotic genes during preimplantation embryo development suggests “alterations in the nuclear-to-cytoplasmic ratios manifested as multinucleation as well as organelle and other cytoplasmic dimorphisms”^88^. These abnormalities further interfere with the developmental potential of the embryo as its transfer may result in low implantation rates^88^. In the apoptotic pathway, the Apaf1 gene activates the effector caspases, thus leading to DNA fragmentation ^30^. Despite the fact that the Apaf1 is a well-elucidated gene in the mammalian apoptotic pathway, the study of Honarpour et al. (2000)^33^ demonstrated that adult Apaf1-deficient male mice had impaired spermatogenesis, resulting in the complete absence of sperm and infertility. However, we cannot confirm the detrimental effect of temperature variation treatments on the relative expression of the Apaf1 gene, since no difference between the treatments and the control group was reported. With the relative gene expression results, we can only confirm that the temperature variation treatment of 37/35.5 °C is indeed detrimental when compared to the other temperature variation treatment proposed (38.5/37 °C). Perhaps apoptosis would be more evident between the study groups if other apoptotic genes were included in this investigation, as studies with knockout mice (caspase-deficient or Apaf1-deficient mice) demonstrated that several other genes might act in the apoptotic pathway and that cell death can be reversed in an Apaf1-independent manner^89^.

The targeted metabolomics of the present study focused on the importance of amino acids during murine embryonic development. The main roles of amino acids in mammalian embryo growth are as protein precursors or as energy sources^90–92^. Nonetheless, as described in the review of Gardner and Harvey (2015)^93^, these metabolites can also be involved in the control of carbohydrate metabolism^94,95^, intracellular signaling^96^ and/or intracellular antioxidants^97^, osmolytes^98^ and chelators^99^.

The catabolism of amino acids during embryo development has been associated with embryo viability, especially in human studie^100,101^. Those studies have demonstrated that the main amino acids consumed by human embryos are arginine, serine, methionine, valine and leucine^93^. In contrast, murine embryos are generally marked by a high consumption of aspartate^90,94^, especially during the blastocyst stage, in which the malate-aspartate shuttle is activated as an alternative way to produce ATP^102^. During the blastocyst stage, amino acids also play an important role in cell differentiation, which will result in the formation of the inner cell mass and the trophectoderm^103,104^.

Our data from the metabolomics of culture media corroborate all other results, in which the T1 group showed adverse effects on embryonic development and quality. Only in this study group were amino acids that suggested a stressed metabolism, such as the presence and higher concentrations of ammonium chloride, identified. Even though the presence of ammonia within the culture media usually indicates the catabolism of amino acids^93^, high concentrations of such metabolites may alter the use of amino acids in the metabolic pathways, resulting in the preeminent production of glutamine^93,105^. The production of glutamine to control ammonia concentrations in the intracellular environment is efficient when levels of stress are low; however, when the levels of stress are persistent, the ammonia concentrations might become toxic, leading to a decrease in embryo viability^93^. In addition to ammonium chloride, aspartic acid, arginine and lysine were also identified in the low-temperature group (T1). Aspartate, as described earlier, is a common amino acid found in mouse culture media due to its role as an energy substrate^90,95^. The amino acids serine and valine, which are then converted into pyruvate and acetyl-CoA, respectively, are also common energy substrates used in the tricarboxylic acid cycle (TCA) of embryonic cells^106^. Additionally, in the malate-aspartate shuttle, aspartate acts as a precursor for other essential amino acids, such as lysine, methionine, threonine and isoleucine^107^. Moreover, aspartate along with arginine plays a role in the urea cycle and consequent process of ammonia detoxification^107^. It was also demonstrated empirically that early human embryos, which were characterized by the consumption of lysine, were associated with the formation of morphologically low-quality blastocysts^107^.

In contrast to the T1 group, the T2 and C groups were generally characterized by metabolites representative of normal embryonic metabolism. Starting with the higher-temperature group (T2), histidine stands out for its role as a paracrine signaling factor in pigs^108^ and human embryos^109^. Another possible role of histidine, when converted to histamine, has been described in the communication between expanded blastocysts and the maternal endometrium, thus influencing implantation^110,111^. Zhao et al*.* (2000)^111^ were able to empirically demonstrate that histidine can originate from uterine epithelial cells in mice, with its highest levels on the day of implantation. The same study was able to demonstrate that preimplantation mouse blastocysts express histamine receptors (H2). Throughout a paracrine interaction, uterine-derived histamine in contact with the H2 receptors initiates the process of implantation^111^. The amino acids glycine, glutamine and alanine are well-elucidated constituents of embryo culture media due to their role as osmolytes, regulating intracellular pH during initial embryonic development^98,112–114^. Proline and leucine, on the other hand, stand out for mouse cell growth and differentiation^104,115–117^. Both amino acids are especially important during the blastocyst stage, stimulating active energetic metabolism and influencing the trophoblast differentiation and consequent implantation^104,115,117,118^. The role of the amino acid ornithine in embryonic metabolism is associated with the function of polyamines. Polyamines are organic molecules composed of more than one amine group, found in both eukaryotic and prokaryotic cells and can be synthesized from the amino acids ornithine, arginine and proline^119^. These organic molecules have several intracellular functions, such as cell growth and proliferation, signal transduction, transcriptional and translational activation, alteration of membrane stability and activation of ion channels^120^. Concerning reproduction, polyamines can impact embryo development, implantation, the embryonic diapause of some animals, the development of the placenta, angiogenesis and fetal development^121^.

Finally, the control group was mainly represented by glutamine, citrulline and methionine. Glutamine, when converted to glutamate and consequent α-ketoglutarate, participates in the production of ATP by embryonic cells through the TCA cycle^122^. The oxidation of glutamine in embryo metabolism has been demonstrated in mouse studies during the early development of culture media^123^. The amino acid glutamine is a common constituent of embryo culture media^106,122^ due to its many roles in intracellular metabolism, such as the catabolism of glucose^124^, as an antioxidant molecule^125^ or as an organic osmolyte^126^. Diversely, citrulline has been associated with the formation of nitric oxide, an important messenger within the cellular microenvironment, during early mouse embryo development^127^. The amino acids citrulline and nitric oxide are formed from arginine throughout the action of the nitric oxide synthase^106,127^. In reproduction, nitric oxide has been demonstrated to participate in signaling processes, such as implantation, pregnancy and labor^128–130^. The metabolite methionine is an amino acid involved in metabolic regulation and nucleotide synthesis, acting mainly during DNA methylation, together with vitamin B12 and folate^131,132^. In addition, methionine, like cysteine, acts as an intracellular antioxidant and is oxidized to methionine sulfoxide from several active species^133^. The oxidative or reducing role of methionine can influence the posttranslational regulation of proteins and their respective functions^134^.

## Conclusions and clinical implications

Overall, our data demonstrated that temperature variation treatments affect the in vitro development of mouse embryos and the quality of blastocysts. Our lower-temperature group or T1 (37/35.5 °C) consistently showed a negative effect on mouse embryo development with “slow” embryo characterization after morphokinetic evaluation and poorly graded blastocysts in the morphological assessment. Following the same pattern, at the molecular level the low-temperature group showed a higher expression of the apoptotic gene Apaf1 in the blastocysts and indicators of a stressed metabolism through the metabolomics of culture media. On the other hand, our higher-temperature group or T2 (38.5/37 °C) showed similar results to the control group, with “normal” cleaving embryos, fairly graded blastocysts and no negative effects at the molecular level regarding stress, apoptosis or metabolism. Further investigations are needed to establish the appropriate temperature variation treatment for human embryos and the appropriate length (hours) of alternation, as well as possible implications on implantation rate and foetus development. Finally, our data are of clinical importance, as “slow” cleavage or poorly graded embryos from patients undergoing IVF treatments may benefit from a higher temperature variation treatment before transfer.

## Supplementary Information

Supplementary Information 1.

Supplementary Video 1.

Supplementary Video 2.
