# Supplementary material for: The effects of temperature variation treatments on embryonic development: a mouse study
Source: Sci Rep. 2022 Feb 15;12:2489. doi: 10.1038/s41598-022-06158-y (PMC8847426; doi:10.1038/s41598-022-06158-y)
Supplement: Supplementary file 1 — Supplementary Information 1. [file 41598_2022_6158_MOESM1_ESM.docx]

**SUPPLEMENTARY MATERIAL**

**The effects of temperature variation treatments on embryonic development: a mouse study**

**Authors:** Dóris Ferreira Moriyama^1^**^*^**, PhD, Dimitra Makri^2^, PhD, Mary-Naya Maalouf^2^, M.Med.Sci, Petra Adamova^2^, M.Med.Sci, Gabrielle Ferrante Alves de Moraes^1^, M.Sci, Marcela de Oliveira Pinheiro^1^, BSc, Danilo Lessa Bernardineli^3^, BSc, Irineu Francisco Delfino Silva Massaia^4^, PhD, Walid E. Maalouf^2^, PhD, Edson Guimarães Lo Turco^1^, PhD.

^1^Department of Surgery, Division of Urology, Human Reproduction Section, Medical School - EPM, Universidade Federal de São Paulo – UNIFESP, São Paulo, Brazil, 04039-060.

^2^Division of Child Health, Obstetrics, and Gynaecology, East Block Queen’s Medical Centre, School of Medicine, University of Nottingham, Nottingham, United Kingdom, NG72UH.

^3^Institute of Physics, Universidade de São Paulo – IF USP, 05508-020.

^4^Faculdade de Ciências Médicas da Santa Casa de São Paulo, São Paulo, Brazil, 01221-020.

**^*^Corresponding author email address:** dorismoriyama@gmail.com

**Table I: Definition of morphokinetic timepoint events from the Consistent annotation for better annotation - A guide on definitions for morphokinetic (Tech-Notes Vitrolife, 2019).**

| **Timepoint** | **Cell stage** | **Criteria** |
| --- | --- | --- |
| **t2, t3, t4, t5, t8** | 2-cell, 3-cell, 4-cell, 5-cell, 8-cell | First frame in which the 2, 3, 4, 5, or 8 cells are clearly separated by membranes accordingly |
| **tC** | compaction | First sign of membrane fusion between the neighbouring blastomeres of the 8-cell stage embryo |
| **t9^+^** | 9^+^ cells | First frame of extrusion of a blastomere from the compacted embryo |
| **tM** | Morula | First sign of fusion of the membranes between neighbouring blastomeres of the 9+ embryo * |
| **tSB** | Small blastocoel | First sign of blastocoel formation |
| **tB** | Blastocyst | Last frame before the embryo starts pushing against the ZP |
| **tEB** | Expanded Blastocyst | First frame in which the ZP thickness is half than that of the B stage |
| **tH** | Hatching | First sign of cells escaping the ZP |

*****formation of a compacted embryo with increased movements within the ZP

**Table II: Calculations of morphodynamic events on embryo development (Adapted from Ciray et al. 2014 and Cetinkaya et al. 2015).**

| **Relative timings** | **Morphodynamic event** | **Calculation** |
| --- | --- | --- |
| **ECC2** | Duration of second cell cycle | t4-t2 |
| **ECC3** | Duration of third cell cycle | t8-t4 |
| **S2** | Synchronization of cell divisions | t4-t3 |
| **S3** | Synchronization of cleavage pattern | t8-t5 |
| **CS2-4** | 2- to 4- cell stage cleavage synchronicity | (t4-t3) / (t4-t2) |
| **CS2-8** | 2- to 8- cell stage cleavage synchronicity | ((t3-t2) + (t5-t4)) / (t8-t2) |
| **CS4-8** | 4- to 8- cell stage cleavage synchronicity | (t8-t5) / (t8-t4) |
| **dB** | Duration of blastulation | tB-tSB |

**Table III: Morphokinetic descriptive data, relative timings and morphodynamical calculations (N=161).**

|  | **T1) 37/35.5°C**  **(N=53)** | **T2) 38.5/37°C**  **(N=53)** | **C) 37°C**  **(N=55)** | ***P*** | **Effect size** | **Power** |
| --- | --- | --- | --- | --- | --- | --- |
| **t2** | 1.61 ± 0.03**^a^** | 1.48 ± 0.03**^b^** | 1.57 ± 0.03**^ab^** | 0.039 | 0.04 | 62% |
|  | 1.54 - 1.69 | 1.41 - 1.56 | 1.50 - 1.63 |  |  |  |
| **t3** | 23.09 ± 0.13^a^ | 20.72 ± 0.20^b^ | 21.19 ± 0.11^b^ | 0.0001 | 0.44 | 100% |
|  | 22.81 - 23.37 | 20.31 - 21.13 | 20.96 - 21.23 |  |  |  |
| **t4** | 23.73 ± 0.16^a^ | 21.40 ± 0.16^b^ | 21.97 ± 0.14^b^ | 0.0001 | 0.42 | 100% |
|  | 23.39 - 24.07 | 21.08 - 21.70 | 21.68 - 22.26 |  |  |  |
| **t5** | 34.36 ± 0.25**^a^** | 31.49 ± 0.27**^b^** | 31.82 ± 0.15**^b^** | 0.0001 | 0.36 | 100% |
|  | 33.85 - 34.87 | 30.95 - 32.04 | 31.50 - 32,14 |  |  |  |
| **t8** | 35.03 ± 0.83**^a^** | 32.00 ± 0.80**^b^** | 33.34 ± 0.18^a^**^b^** | 0.007 | 0.06 | 81% |
|  | 33.35 - 36.70 | 30.39 - 33.61 | 32.96 - 33.72 |  |  |  |
| **ECC2** | 22.11 ± 0.15**^a^** | 19.92 ± 0.14**^b^** | 20.40 ± 0.14**^b^** | 0.0001 | 0.43 | 100% |
|  | 21.80 - 22.80 | 19.62 - 20.21 | 20.12 - 20.69 |  |  |  |
| **ECC3** | 11.29 ± 0.82 | 10.59 ± 0.79 | 11.37 ± 0.14 | 0.656 | 0.005 | 11% |
|  | 9.63 - 12.95 | 9.00 - 12.18 | 11.08 - 11.65 |  |  |  |
| **S2** | 0.64 ± 0.08 | 0.68 ± 0.16 | 0.78 ± 0.08 | 0.692 | 0.005 | 10% |
|  | 0.48 - 0.80 | 0.35 - 1.07 | 0.60 - 0.95 |  |  |  |
| **S3** | 1.53 ± 0.11 | 1.25 ± 0.08 | 1.52 ± 0.13 | 0.526 | 0.008 | 15% |
|  | 1.29 - 1.77 | 1.08 - 1.43 | 1.25 - 1.79 |  |  |  |
| **CS24** | 0.02 ± 0.003 | 0.03 ± 0.008 | 0.03 ± 0.003 | 0.505 | 0.009 | 16% |
|  | 0.02 - 0.03 | 0.01 - 0.04 | 0.02 - 0.04 |  |  |  |
| **CS28** | 0.82 ± 0.11 | 0.84 ± 0.08 | 0.92 ± 0.005 | 0.640 | 0.006 | 12% |
|  | 0.59 - 1.05 | 0.66 - 1.02 | 0.91 - 0.93 |  |  |  |
| **CS48** | 0.15 ± 0.02 | 0.13 ± 0.02 | 0.13 ± 0.009 | 0.741 | 0.004 | 9% |
|  | 0.09 - 0.20 | 0.08 - 0.18 | 0.11 - 0.15 |  |  |  |
| **tM** | 47.74 ± 1.11 | 45.01 ± 0.39 | 45.12 ± 1.01 | 0.057 | 0.03 | 56% |
|  | 45.51 - 49.98 | 44.22 - 45.80 | 43.09 - 47.16 |  |  |  |
| **tSB** | 61.39 ± 1.44**^a^** | 53.92 ± 0.51**^b^** | 56.78 ± 0.38**^b^** | 0.0001 | 0.17 | 100% |
|  | 58.48 - 64.30 | 52.88 - 54.96 | 56.02 - 57.55 |  |  |  |
| **tB** | 66.00 ± 1.56**^a^** | 58.23 + 0.56**^b^** | 61.43 ± 0.50**^b^** | 0.0001 | 0.16 | 99% |
|  | 62.87 - 69.14 | 57.09 - 59.36 | 60.42 - 62.34 |  |  |  |
| **tEB** | 69.84 ± 2.74 | 65.26 ± 0.65 | 68.06 ± 0.60 | 0.147 | 0.02 | 39% |
|  | 64.34 - 75.34 | 63.95 - 66.58 | 66.85 - 69.26 |  |  |  |
| **dB** | 4.61 ± 0.38 | 4.30 ± 0.29 | 4.64 ± 0.30 | 0.727 | 0.004 | 10% |
|  | 3.84 - 5.37 | 3.70 - 4.90 | 4.03 - 5.25 |  |  |  |

Values represented as mean+standard error and 95% Confidence Interval lower-upper bound, difference between groups represented by ^a,b^ or ^ab^.

**Table IV: Relative gene expression descriptive results (N=83)**

| **Relative gene expression** | **T1) 37/35.5°C**  **(N=24)** | **T2) 38.5/37°C**  **(N=30)** | **C) 37°C**  **(N=29)** | ***P*** | **Effect size** | **Power** |
| --- | --- | --- | --- | --- | --- | --- |
| **ΔCT Igf2/Rpl5** | 4.35 ± 0.19 | 4.39 ± 0.095 | 4.45 ± 0.16 | 0.894 | 0.003 | 67% |
|  | 3.94 - 4.75 | 4.19 - 4.59 | 4.11 - 4.79 |  |  |  |
| **ΔCT Bax/Rpl5** | 3.04 ± 0.06 | 2.99 ± 0.07 | 3.03 ± 0.05 | 0.837 | 0.004 | 77% |
|  | 2.91 - 3.17 | 2.83 - 3.15 | 2.92 - 3.15 |  |  |  |
| **ΔCT Apaf1/Rpl5** | 7.19 ± 0.16**^a^** | 7.93 ± 0.16**^b^** | 7.52 ± 0.11^a^**^b^** | **0.003** | 0.13 | 87% |
|  | 6.86 - 7.52 | 7.59 - 8.28 | 7.29 - 7.76 |  |  |  |

Values represented as mean ± standard error and 95% Confidence Interval lower-upper bound, difference between groups represented by ^a,b^ or ^ab^.

**Table V: Metabolomics data (N=81)**

| **Metabolomics data: mean±SD** | | **Study Groups** | | |
| --- | --- | --- | --- | --- |
| **Abreviations** | **Metabolites** | **T1** | **T2** | **C** |
| **1-MHIS** | 1-Methyl-L-Histidine | 0 | 0 | 0 |
| **3-MHIS** | 3-Methyl-L-Histidine | 0 | 0 | 0 |
| **ALA** | L-Alanine | 1.74 ± 1.67 | 175.14 ± 124.95 | 1.71 ± 1.90 |
| **AMM** | Ammonium Chloride | 1.19 ± 4.08 | 716.06 ± 1.03 | 455.10 ± 6.56 |
| **ANS** | Anserine | 2.69 ± 4.49 | 318.34 ± 2.11 | 3.12 ± 3.57 |
| **ARG** | L-Arginine | 0 | 0 | 0 |
| **ASN** | L-Asparagine | 0 | 0 | 0 |
| **ASP** | L-Aspartic acid | 3.88 ± 379.19 | 3.77 ± 7.24 | 3.77 ± 3.78 |
| **B-ALA** | β-Alanine | 0 | 0 | 0 |
| **CAR** | L-Carnosine | 0 | 0 | 0 |
| **CITR** | Citrulline | 0 | 0.36 ± 1.19 | 0.49 ± 0.87 |
| **CYS** | Cystine | 2.43 ± 2.61 | 2.54 ± 1.92 | 2.50 ± 2.72 |
| **CYSTH** | cystathionine | 0.08 ± 0.21 | 0.19 ± 0.25 | 0.07 ± 0.20 |
| **ETHAMN** | Ethanolamine | 0 | 0 | 0 |
| **GLN** | Glutamine | 138.92 ± 1.35 | 1.40 ± 8.58 | 1.44 ± 1.84 |
| **GLU** | L-Glutamic acid | 3.48 ± 3.75 | 3.63 ± 2.46 | 3.51 ± 3.71 |
| **GLY** | Glycine | 309.36 ± 356.64 | 3.39 ± 2.24 | 3.37 ± 3.64 |
| **HIS** | L-Histidine | 6.29 ± 6.76 | 6.97 ± 4.57 | 6.68 ± 7.45 |
| **HOMOCYS** | Homocystine | 0 | 0 | 0 |
| **HYLYS 1** | D-allohydroxylysine | 0 | 0 | 0 |
| **HYLYS 2** | L-allohydroxylysine | 0 | 0 | 0 |
| **ILE** | L-Isoleucine | 1.30 ± 1.27 | 143.81 ± 9.16 | 1.37 ± 1.49 |
| **LEU** | L-Leucine | 1.42 ± 146.80 | 1.45 ± 8.55 | 1.42 ± 1.42 |
| **LYS** | L-Lysine | 1.41 ± 1.30 | 1.40 ± 7.67 | 1.36 ± 1.43 |
| **MET** | L-Methionine | 2.55 ± 4.68 | 29.52 ± 8.34 | 3.51 ± 8.32 |
| **NLEU** | Norleucine | 0 | 0 | 0 |
| **ORN** | L-Ornithine | 0.20 ± 0.39 | 0.78 ± 0.47 | 0.10 ± 0.34 |
| **PEA** | Phosphoethanolamine | 0 | 0 | 0 |
| **PHE** | Phenylalanine | 6.78 ± 6.76 | 7.19 ± 3.95 | 7.15 ± 7.17 |
| **PHSER** | Phosphoserine | 0 | 0 | 0 |
| **PRO** | L-Proline | 0 | 0 | 0 |
| **SARC** | L-Sarcosine | 0 | 0 | 0 |
| **SER** | Serine | 383.55 ± 4.59 | 3.63 ± 2.22 | 3.55 ± 3.69 |
| **TAUR** | Taurine | 0 | 0 | 0 |
| **THR** | L-Threonine | 1.40 ± 3.13 | 145.26 ± 7.92 | 1.42 ± 1.50 |
| **TRYP** | Tryptophan | 2.03 ± 2.87 | 67.13 ± 0.77 | 6.62 ± 1.62 |
| **TYR** | Tyrosine | 79.67 ± 9.85 | 74.28 ± 5.66 | 7.39 ± 7.29 |
| **UREA** | Urea | 0 | 0 | 0 |
| **VAL** | Valine | 1.45 ± 1.35 | 1.41 ± 7.50 | 1.33 ± 1.53 |

Values represented as mean ± standard deviation. Unit of measurement is micromolar [μM].

**Supplementary Video A: Early embryo development (from t2 to t8)**

Video A shows clearly the differences between groups regarding the early embryonic morphokinetics. For all parameters, T1 group showed a slower development when compared to both T2 and C groups.

**Supplementary Video B: Embryo development post-compaction (from tSB to tEB)**

Video B shows clearly the differences between groups regarding embryonic morphokinetics post-compaction. For all parameters, T1 group showed a slower development when compared to both T2 and C groups.
